# Paramutation-Like Behavior of Genic piRNA-Producing Loci in *Drosophila virilis*

**DOI:** 10.3390/ijms26094243

**Published:** 2025-04-29

**Authors:** Alina V. Bespalova, Dina A. Kulikova, Elena S. Zelentsova, Alexander P. Rezvykh, Iuliia O. Guseva, Ana P. Dorador, Mikhail B. Evgen’ev, Sergei Y. Funikov

**Affiliations:** 1 Engelhardt Institute of Molecular Biology, Russian Academy of Sciences, 119991 Moscow, Russia; 2 Koltzov Institute of Developmental Biology, Russian Academy of Sciences, 119334 Moscow, Russia; 3 Moscow Center for Advanced Studies, Kulakova Str. 20, 123592 Moscow, Russia; 4 Howard Hughes Medical Institute, Department of Cell Biology, Harvard Medical School, Boston, MA 02115, USA

**Keywords:** genic piRNAs, piRNA clusters, transposable elements, maternal effect, paramutation, telomere, *Drosophila virilis*

## Abstract

Piwi-interacting RNAs (piRNAs) play a crucial role in silencing transposable elements (TEs) in the germ cells of Metazoa by acting as sequence-specific guides. Originating from distinct genomic loci, called piRNA clusters, piRNA can trigger an epigenetic conversion of TE insertions into piRNA clusters by means of a paramutation-like process. However, the variability in piRNA clusters’ capacity to induce such conversions remains poorly understood. Here, we investigated two *Drosophila virilis* strains with differing capacities to produce piRNAs from the subtelomeric *RhoGEF3* and *Adar* gene loci. We found that active piRNA generation correlates with high levels of the heterochromatic mark histone 3 lysine 9 trimethylation (H3K9me3) over genomic regions that give rise to piRNAs. Importantly, the maternal transmission of piRNAs drives their production in the progeny, even from homologous loci previously inactive in piRNA biogenesis. The *RhoGEF3* locus, once epigenetically converted, maintained enhanced piRNA production in subsequent generations lacking the original allele carrying the active piRNA cluster. In contrast, piRNA expression from the converted *Adar* locus was lost in offspring lacking the inducer allele. The present findings suggest that the paramutation-like behavior of piRNA clusters may be influenced not only by piRNAs but also by structural features and the chromatin environment in the proximity to telomeres, providing new insights into the epigenetic regulation of the Drosophila genome.

## 1. Introduction

In animals, a distinct class of small non-coding RNAs called Piwi-interacting RNAs (piRNAs) plays a crucial role in the silencing of transposable elements (TEs) (reviewed in [1,2]). In Drosophila, piRNAs repress TEs through two different mechanisms. Within the nucleus, piRNAs in complexes with Piwi protein are responsible for the deposition of H3K9me3 mark (trimethylation of lysine 9 on histone 3) on homologous transposon targets [3,4,5,6,7]. In the cytoplasm, two additional proteins, Aub and Ago3, are guided by the associated piRNAs to cleave homologous TE transcripts utilizing their endonuclease activity [8,9]. The cleavage of complementary transcripts by Aub and Ago3 also results in the biogenesis of new piRNAs by a process known as the ping-pong amplification cycle [10].

In most animals, a specific number of genomic regions termed piRNA clusters have been identified as the licensed sources responsible for producing the majority of piRNAs (reviewed in [11]). In the Drosophila germline, dual-strand piRNA clusters are the predominant form of piRNA source [10,12,13,14,15]. Most piRNA clusters are located in the pericentromeric and subtelomeric regions of chromosomes [10]. These genomic regions are mainly composed of repetitive sequences, including the remnants of various TEs and satellite DNA, and are enriched in heterochromatin protein 1 (HP1) and its germline paralog Rhino, which are capable of binding to di- or trimethylated H3K9 via their chromodomains [16,17,18]. Moreover, H3K9me3 mark, which is generally associated with constitutive heterochromatin, and Rhino are required for the transcription of piRNA clusters in Drosophila [19,20,21,22,23,24].

What determines Rhino binding and the identification of piRNA clusters in the genome remains one of the major questions in the field. In fact, it has been demonstrated that any sequence that is inserted into piRNA clusters can be processed into piRNAs [25]. Studies by Akkouche et al. and Le Thomas et al. have underscored the pivotal role of the maternal deposition of piRNAs through the female germline in the establishment of piRNA clusters in subsequent generations [21,26,27]. Furthermore, serving as inherited epigenetic signaling molecules, piRNAs are also capable of converting previously naïve homologous loci into a piRNA cluster. These findings underlie the molecular basis of paramutation phenomena in Drosophila [28]. Paramutation describes an interaction between two alleles of a locus in which one allele (paramutagenic) initiates an inherited epigenetic conversion of the other allele (paramutable) without altering the DNA sequence (reviewed in [29,30]). The paramutation model in Drosophila is characterized by the maternal transmission of piRNAs, which serve to initiate the production of piRNAs from homologous loci *in trans* [21,28]. However, given the limited number of paramutation models, it remains unclear whether piRNA production is sufficient to cause the epigenetic conversion of the naïve homologous loci via paramutation.

What factors trigger the de novo formation and maintenance of piRNA source loci? It has been shown that the piRNA silencing of TEs can nucleate heterochromatin formation at euchromatic TE insertions [31,32]. Furthermore, de novo euchromatic TE insertions can trigger the formation of novel dual-strand piRNA clusters [31,33,34,35]. The effect of epigenetic TE silencing can spread from TE insertions, thereby affecting the expression of adjacent genes [7,32,36,37,38]. Thus, in rare cases, the piRNA machinery may mistakenly target non-TE sequences, thereby initiating the formation of piRNA clusters encompassing protein-coding genes [13,39,40]. A shift from canonical mRNA processing to piRNA biogenesis at the *Mps1* and *cdi* loci serves as an example of such “unintended” regulation in *Drosophila melanogaster* and *Drosophila virilis*, respectively [27,41]. Furthermore, it has been demonstrated that piRNAs generated from the *cdi* locus in *D. virilis* have the capacity to trigger the conversion of the homologous naïve allele, thereby exhibiting paramutation-like behavior [42].

The present study builds on our previous findings of *D. virilis* strains that exhibited a differential capacity to generate piRNAs from several genomic loci [13]. We characterized several protein-coding genes, including *RhoGEF3*, *Adar*, and *OtopLb*, that show high levels of sense and antisense piRNA production in the ovaries and testes of the *D. virilis* strain *140* but not in the gonads of strain *9*. We found that the ovarian piRNA biogenesis at these specific gene loci is correlated with the enrichment of heterochromatic H3K9me3 mark. It has been determined that the *RhoGEF3* and *Adar* loci are located in the subtelomeric regions of chromosomes 3 and X, respectively. A detailed analysis of the structural features of the *RhoGEF3* locus in both studied strains revealed the insertion of the LTR retroelement *Gypsy1* upstream of the *RhoGEF3* promoter region in strain *140* as a potential determinant of the genic piRNA production. We also show that maternally deposited piRNAs derived from the *Adar* locus result in the silencing of *Adar* gene expression in the progeny. Strikingly and unexpectedly, the high level of piRNAs targeting *RhoGEF3* was shown to be positively correlated with the expression of *RhoGEF3* mRNA. Importantly, we identified the allelic origin of piRNAs in the hybrid genome and unambiguously demonstrated that the maternal deposition of piRNAs leads to a higher production of piRNAs at the previously inactive homologous loci. Finally, we monitored these piRNA clusters for paramutagenic activity. The results indicated that piRNA production from the *RhoGEF3* locus persists in the progeny in the absence of the original allele carrying the active piRNA cluster. Conversely, the expression of piRNAs from the *Adar* locus was lost in offspring devoid of the original inducer allele. Our results demonstrate that the paramutation-like behavior of piRNA clusters is shaped not only by piRNAs but also by the structural characteristics of relevant chromosomal regions.

## 2. Results

### 2.1. Gene Targeting by piRNAs Exhibits Sexual Dimorphism in Drosophila virilis

**Figure 1:**
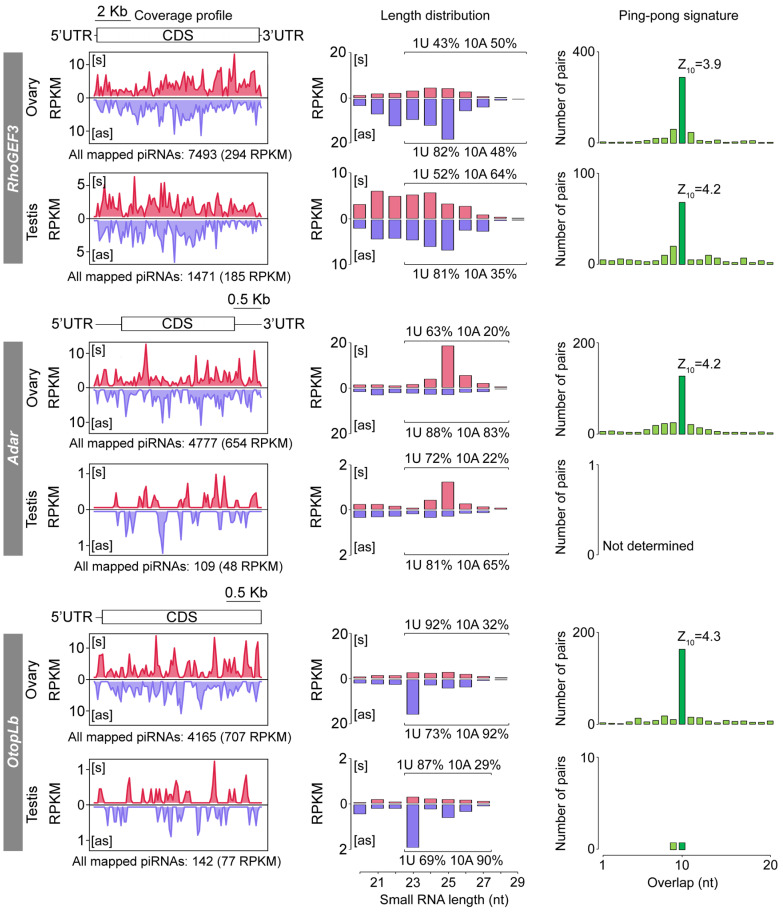
Analysis of gene-derived piRNAs in the ovaries and testes of *D. virilis* strain *140*. The **left** panels show profiles of mapped piRNAs (23–29 nt) for *RhoGEF3*, *Adar*, and *OtopLb* transcripts in both sexes. Only uniquely aligned piRNA reads with up to 3 mismatches are shown. Expression values were normalized to the number of reads per kilobase per million mapped reads (RPKM). Transcripts regions, including coding sequences (CDS), 5′- and 3′-untranslated regions (UTRs), are shown above the plots. The total number of uniquely aligned piRNAs is displayed below the plots. The **middle** panels show the size distribution of uniquely mapped small RNAs (20–29 nt) with up to 3 mismatches to each gene transcript. Nucleotide biases, including 1U and 10A (percentage), are shown for both sense and antisense piRNA mappers (23–29 nt). The **right** panels are the number of complementary piRNA pairs showing enrichment for a 10 nt overlap between sense and antisense sRNAs (i.e., the ping-pong signature). Ping-pong Z-scores are shown above the plots. S (red) and AS (blue) denote small RNAs mapped in the sense and antisense orientations, respectively.

In search of gene targeting piRNAs, we re-examined small RNA libraries obtained previously from the ovaries and testes of several *D. virilis* strains [13]. An analysis of piRNAs (23–29 nt) uniquely aligned to the annotated *D. virilis* gene transcripts revealed high levels of sense and antisense piRNA expression originating from three transcripts of protein-coding genes, *RhoGEF3* (294 normalized piRNAs, reads per kilobase per million mapped reads (RPKM)), *Adar* (654 RPKM), and *OtopLb* (707 RPKM), in the ovaries of strain *140* (Figure 1). These piRNAs cover the entire transcript, including the 5′- and 3′-untranslated regions (UTRs), as well as coding sequences (CDS) (Figure 1). The *RhoGEF3* gene encodes a member of the Rho GTPases, a major branch of the Ras superfamily of small GTPases, which regulate actin cytoskeleton organization [43]. *Adar* encodes an RNA-editing enzyme that catalyzes the deamination of adenosine nucleotides to inosines on double-stranded RNAs [44]. *OtopLb* encodes one of the Otopetrin proteins, which function as proton channels conserved among vertebrates and invertebrates [45].

Interestingly, the expression of piRNA targeting these gene transcripts also occurs in the male gonads (Figure 1). We observed a high level of piRNA expression targeting *RhoGEF3* mRNA in testes, which is only ~1.5-fold less in comparison to the ovarian tissue (Figure 1). However, piRNA production targeting *Adar* and *OtopLb* mRNAs is ~10-fold lower in testes than in ovaries (Figure 1). Despite this difference in abundance, the presence of these piRNAs in both gonads suggests a shared gene targeting program in the gonads of both sexes in *D. virilis*.

piRNA reads mapped in the antisense orientation to mRNA sequences show a strong bias towards U at the first nucleotide, a feature of *bona fide* piRNAs [10], ranging from 69% for *OtopLb* in testis to 88% for *Adar* in ovaries (Figure 1). Moreover, piRNAs from all three analyzed gene transcripts show a ping-pong signature, i.e., enrichment for a 10-nt overlap between 5′ ends of complementary piRNA pairs, with the exception of the *Adar* and *OtopLb* genes in testes (Figure 1). Probably, the number of mapped piRNAs was insufficient to identify overlapping piRNA pairs for *Adar* and *OtopLb* in testes. Notably, piRNAs mapped in sense orientation to *RhoGEF3* transcript demonstrate a lower 1U bias but a greater preference for A at the tenth nucleotide compared to their antisense counterparts (Figure 1). The observed variations in nucleotide bias for *RhoGEF3* most likely indicate that distinct DNA strands give rise to piRNA precursor transcripts that are processed into 1U and 10A piRNAs.

Thus, we demonstrated that the protein-coding genes *RhoGEF3*, *Adar*, and *OtopLb* are targeted by sense and antisense piRNAs in the ovaries and testes of *D. virilis* strain *140*.

### 2.2. Similar Genomic Loci Exhibit Divergent Capacity to Generate piRNAs and Form Dual-Strand piRNA Clusters in D. virilis Strains

**Figure 2:**
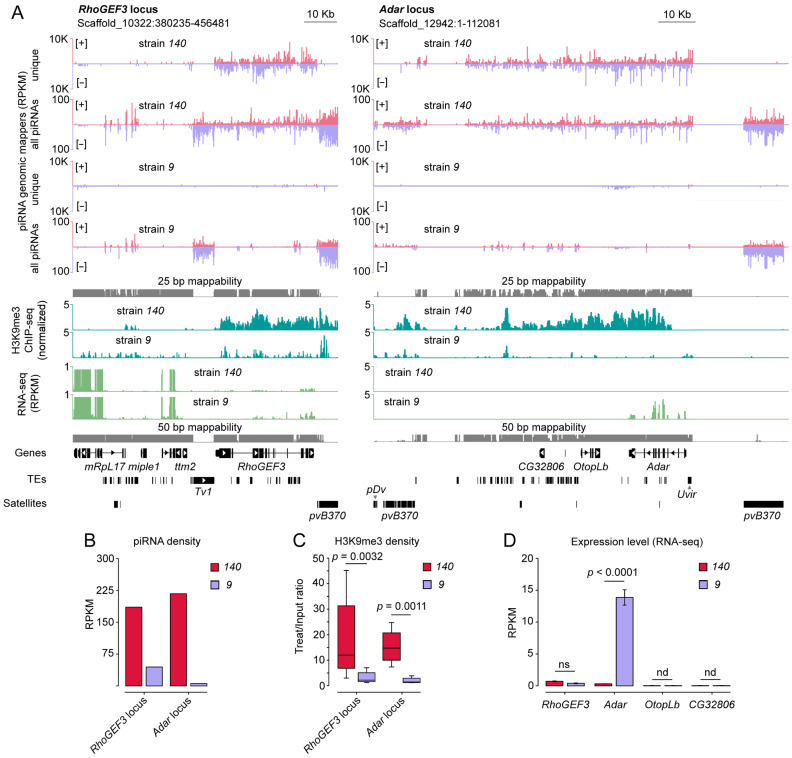
Profiles of piRNAs, H3K9me3 heterochromatic mark, and mRNAs at the *RhoGEF3* and *Adar* loci in strains *140* and *9*. (**A**) piRNAs (small RNA-seq), H3K9me3 (ChIP-seq), and mRNAs (poly(A) RNA-seq) densities at the *RhoGEF3* and *Adar* loci in ovaries of the two *D. virilis* strains. Unique and all piRNA mappers are shown with respect to their mapping orientation to the [+] and [−] DNA strand. The resulting H3K9me3 profiles were calculated by subtracting the input samples from treat ChIP samples, followed by normalization using the RPGC method (number of reads per bin/scaling factor for 1× average coverage of the genome). Gene names are given according to the names of their orthologues in *D. melanogaster*. Unique mappability tracks for 25- and 50-bp-long reads are shown in gray. (**B**) Difference in the expression of piRNA clusters in the two *D. virilis* strains. Only unique piRNA mappers were used to estimate the overall piRNAs mapped to the specified genomic loci. (**C**) Enrichment of the H3K9me3 mark within the defined regions of piRNA clusters in strains *140* and *9* as measured by ChIP-seq. Only regions that enriched in treat vs. input samples with *p*-adj < 0.05 were used. *p*-values were calculated using the Mann–Whitney U test. (**D**) Expression levels of *RhoGEF3*, *Adar*, *OtopLb*, and *CG32806* located within piRNA clusters. RNA-seq reads mapped on gene exons were normalized to transcript length as well as sequencing depth (RPKM, reads per kilobase per million mapped reads). *p*-values represent *p*-adjusted values calculated by differential expression analysis. ns indicates non-significant expression differences; nd means expression was not determined. K indicates thousands.

Given the unique correspondence of piRNAs to gene transcripts, we hypothesized that the *RhoGEF3*, *Adar*, and *OtopLb* gene loci themselves are the sources of piRNA production. To characterize these genomic loci, we uniquely mapped piRNAs (23–29 nt) to the reference genome of *D. virilis* (release 1.06, fetched from https://flybase.org/ Accessed on 15 March 2020) assembled and annotated in the 12 Drosophila genomes project [46]. We observed that genomic regions harboring the analyzed genes produce piRNAs from both DNA strands in strain *140* but not in strain *9*, indicating the presence of differentially expressed dual-strand piRNA clusters in *D. virilis* (Figure 2A,B). According to the unique piRNA mapping profile, the boundaries of piRNA production are predominantly confined to the *RhoGEF3* gene region (Figure 2A). Note, only a small number of piRNAs were aligned to the *ttm2* gene, which is located next to the *RhoGEF3* gene. The *Adar* and *OtopLb* genes are located in close proximity to each other within a single dual-strand piRNA cluster, the boundaries of which extend beyond the loci of these genes (Figure 2A). We observed that the piRNA profile in *Adar* locus covers an additional protein-coding gene *CG32806* in the ovaries and testes of strain *140* (Figure 2A; Supplementary Figure S1). The counting of piRNA reads aligned to *CG32806* transcripts indicates the generation of sense and antisense piRNA with intrinsic nucleotide bias as well as ping-pong processing in ovaries (Supplementary Figure S2). Notably, piRNA production occurs unevenly in different regions of the same piRNA cluster at the *Adar* locus. Based on the number of piRNAs mapped to gene transcripts from this locus, the region harboring *Adar* gene (654 RPKM) is more actively generates piRNAs than the region where *CG32806* is located (143 RPKM) (Figure 1, Figure 2A, and Figure S2). Moreover, piRNA profiling of these two piRNA clusters revealed similar patterns in both ovaries and testes in terms of the boundaries of piRNA production (Figure 2A and Figure S1). However, as mentioned in the previous section, there is a difference in the level of piRNA expression, which is stronger in female gonads (Figure 1 and Figure S2).

In the reference genome used for NGS data mapping, the *RhoGEF3* gene is surrounded by repeats, including an insertion of the LTR retroelement, *Tv1*, and satellite *pvB370* upstream and downstream of the *RhoGEF3* gene, respectively (Figure 2A). Note that the genomic region upstream of the *Tv1* insertion encompasses approximately 300 Kb, which are densely packed with genes. The piRNA cluster at the *Adar* locus contains a *Uvir* retroelement insertion upstream of the *Adar* gene. In addition, arrays including satellites *pDv* and *pvB370* are located at the boundaries of the unique piRNA mapping. In order to provide a more detailed characterization of the boundaries of the defined piRNA clusters, we also examined non-unique piRNA mapping profiles at the two loci. The results demonstrated that these repeats are also targets for piRNAs in the *D. virilis* genome (Figure 2A). Consequently, it can be hypothesized that the boundaries of the piRNA cluster at the *Adar* locus could be extended to encompass regions where satellite arrays are located. Nevertheless, in the case of the *RhoGEF3* locus, the satellite array is located only downstream of the *RhoGEF3* gene; whereas, the upstream boundary is restricted by the *Tv1* insertion. It is noteworthy that there are gaps located at the *Adar* locus in the piRNA cluster, which are devoid of aligned piRNA reads (Figure 2A). This probably indicates structural genomic differences between the reference and analyzed fly strains.

The heterochromatic mark H3K9me3 is known to be required for the production of piRNAs in Drosophila [20,23]. To determine whether piRNA-producing loci have different chromatin states in the strains studied, we profiled H3K9me3 in the ovaries. We performed chromatin immunoprecipitation followed by sequencing (ChIP-seq) for strain *140* and re-analyzed ChIP-seq data for strain *9* published previously [27]. We demonstrated that genomic regions generating genic piRNAs exclusively in strain *140* are significantly more enriched in the H3K9me3 mark in this strain compared to strain *9* (*p* < 0.001 for both *RhoGEF3* and *Adar* loci, Mann–Whitney U test) (Figure 2A,C). Therefore, the activity of both described piRNA clusters in *D. virilis* correlates with high levels of the H3K9me3 mark.

To understand how H3K9me3 occupancy and piRNA production affect gene expression, we performed poly(A) RNA-seq using the ovaries of both fly strains. We showed that high levels of piRNA production and H3K9me3 occupancy correlate with a significant decrease in the expression of *Adar* mRNA in the ovaries of strain *140* compared to strain *9* (~12-fold decrease, *p*-adj < 0.0001) (Figure 2D). In contrast, we failed to detect a significant difference between the expression levels of *RhoGEF3* in strains *140* and *9*, which we attribute to low baseline expression in these tissues (Figure 2D). In agreement with the tissue expression profile of *D. melanogaster* performed during the modENCODE project [47], *OtopLb* and *CG32806* genes are not expressed in Drosophila ovaries (Figure 2D). Thus, it is not possible to assess the effect of piRNA generation and H3K9me3 enrichment on expression of these genes in ovarian tissue.

Overall, we characterized the genomic loci *RhoGEF3* and *Adar*, which show different capacities to produce piRNAs in two *D. virilis* strains studied. The expression of piRNAs originating from two genomic loci correlates with the high occupancy of heterochromatic H3K9me3 mark, indicating that these loci represent actively transcribed dual-strand piRNA clusters in strain *140* but not in strain *9*. We also found that *Adar* mRNA expression was decreased in strain *140* compared to strain *9*, suggesting a link between piRNA cluster activity and the downregulation of a correspondent gene expression. However, this correlation was not observed for the *RhoGEF3* gene.

### 2.3. LTR Retroelement Insertion Predisposed the Activity of the RhoGEF3 Locus as a piRNA Cluster

To assess the structural differences that determine the production of piRNAs in one fly strain but not in another, we performed the long-read DNA sequencing of strain *140* to generate a whole-genome assembly. The genome assembly of strain *9* was published previously (GenBank accession number GCA_016920725.1) [48]. Although the genome assembly of strain *140* resulted in a longer contigs length (N50 = 27.3 Mb) than strain *9* (N50 = 16.9 Mb), the overall assembly length in this strain was shorter (strain *140*: ~168 Mb; strain *9*: ~182 Mb) (Supplementary Table S1). Importantly, the BUSCO percentages of complete genes were close between the assemblies (99.5% for strain *9* and 99.6% for strain *140*) indicating comparable completeness of the genomes (Supplementary Table S1).

Based on the assignment of specific marker genes to the genomic scaffolds made by Schaeffer et al. [49], the genome assemblies of the studied strains, *140* and *9*, as well as the currently available chromosome assembly of another *D. virilis* strain, *160* (GenBank: GCA_007989325.2) [50], both *RhoGEF3* and *Adar* loci are located in the subtelomeric regions of chromosome 3 (Muller D) and X chromosome (Muller A), respectively.

**Figure 3:**
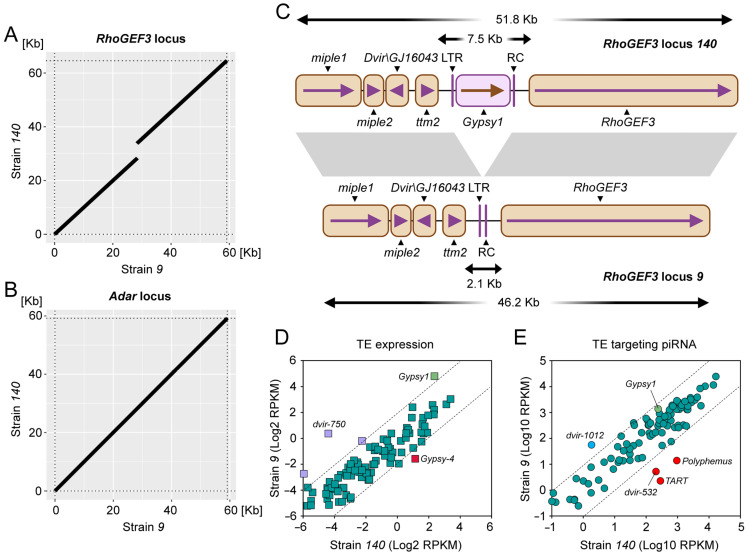
Comparative sequence analysis of the *RhoGEF3* and *Adar* loci in the two studied *D. virilis* strains. (**A**,**B**) Dot plots depicting the gap between the *RhoGEF3* loci and the identity of the *Adar* loci in strains *140* and *9*. Up to 10% sequence divergence was allowed in the sequence alignment. (**C**) Schematic representation of the alignment of *RhoGEF3* loci in strains *140* and *9*. *Gypsy1*—full insertion of the Gypsy superfamily TE (5403 bp), LTR—remnant insertion of LTR retrotransposon (87 bp), RC—remnant insertion of rolling circle TE (72 bp). Areas colored in gray demonstrate overall sequence similarity. Gene names are given according to the names of their orthologues in *D. melanogaster*, except for *Dvir\GJ16043*, which has no orthologue in *D. melanogaster*. (**D**) Scatter plot demonstrating TE expression levels in the ovaries of strains *140* and *9* as calculated by RNA-seq. The diagonal dotted line indicates ~4-fold levels of difference. (**E**) Scatter plot representing the results of the pairwise comparison of normalized piRNA expression (23–29 nt) targeting TEs in the ovaries of strains *140* and *9*. Diagonal dotted line indicates ~10-fold levels of difference. All TEs that exceed the 10-fold line (piRNA) or 4-fold line (RNA-seq) are marked with red and blue dots, indicating that expression levels are greater in strains *140* or *9*, respectively.

To perform the comparative analysis of the *RhoGEF3* and *Adar* loci, we first uniquely aligned ovarian piRNAs from strains *140* and *9* to the corresponding genomic assemblies. We then extracted DNA sequences with up to 10 Kb extensions, depending on the constraints of the current assemblies, upstream and downstream from the boundaries of the piRNA mapping profile of the *RhoGEF3* and *Adar* loci. Next, we performed pairwise alignment of extracted sequences and found the gap with a length of ~5.4 Kb in the *RhoGEF3* locus (Figure 3A). The examination of structural variations of the *Adar* locus did not reveal significant differences between the two strains, except for 69 bp indel (insertion/deletion) in the intergenic region between the *Adar* and *OtopLb* genes, as well as several short indels no more than 5–6 bp long (Figure 3B).

A detailed analysis of the gap found between *RhoGEF3* loci in the studied fly strains revealed the full-length insertion of a LTR retroelement *Gypsy1* upstream of the *RhoGEF3* gene in strain *140* (Figure 3C). *Gypsy1* belongs to the Gypsy superfamily and is one of the most active TEs in *D. virilis* in terms of full-length copy number in the genome, TE expression as well as targeting by piRNAs (Figure 3D,E and Figure S3). It is noteworthy that the number of genomic insertions and the expression level of *Gypsy1* are higher in strain *9* in comparison to strain *140* (Figure 3D and Figure S3). However, the expression levels of piRNAs targeting *Gypsy1* were comparable in both *D. virilis* strains (Figure 3E).

As demonstrated in the preceding section, the *RhoGEF3* locus encompasses *pvB370* arrays downstream of the *RhoGEF3* gene. In addition, the piRNA cluster at the *Adar* locus is flanked by *pbB370* arrays (Figure 2A). An analysis of these loci for variations in the structure of satellite arrays using the assembled genomes of the strains studied revealed that *pvB370* array is located towards the telomere of the chromosome and is ~3-fold shorter at the *RhoGEF3* locus in the genome of strain *140* compared to the genome of strains *9* (~20 Kb in strain *140* and ~63 Kb in strain *9*) (Supplementary Figure S4). Furthermore, in strain *140*, but not in strain *9*, the *Penelope* retroelement insertion is located between the *pvB370* arrays.

An analysis of the *Adar* locus confirmed that this region lies between two arrays of *pDv* and *pvB370*, and the *Uvir* retroelement was found to be located upstream of the *Adar* gene towards the telomere in the genome of strain *9* (Supplementary Figure S4). Importantly, consistent with previous analysis, sequences located within the satellite arrays at the Adar locus showed no significant differences between the strains studied. However, the *Adar* gene has neither the *pvB370* array nor the first exon pair according to the strain *140* genome assembly (Supplementary Figure S4). These data probably indicate that the subtelomeric region of chromosome X in strain *140* may not be completely assembled.

Summarizing the obtained results, we suggest that the insertion of LTR retroelement followed by its piRNA-mediated silencing determines the heterochromatic state of the *RhoGEF3* locus in strain *140*. The enrichment of H3K9me3 predisposes this locus to the eventual formation of the dual-strand piRNA cluster in the germ cells. In addition, the satellite array downstream of the *RhoGEF3* gene is shorter in strain *140* than in strain *9*. By analyzing the structure of the *Adar* locus between the two strains, we failed to detect any unambiguous genetic determinants in the current genome assembly that could shape the *Adar* locus to operate as a piRNA cluster. However, our analysis of the *Adar* locus is limited to the current genome assembly.

### 2.4. Maternal Transmission of piRNAs to RhoGEF3 and Adar Loci Drives Their Production in the Progeny

To further study the role of maternal deposition in producing piRNAs in the next generation, we analyzed the expression of piRNAs in the progeny of reciprocal hybrids involving strains *140* and *9*. The progeny of the cross between strain *140* females and strain *9* males was designated as MD (maternal deposition), indicating that piRNAs are inherited from *140* mothers. The opposite cross involving strain *9* females and strain *140* males was designated as NMD (no maternal deposition), the offspring inherit only chromosomes from *140* mothers but not piRNAs.

**Figure 4:**
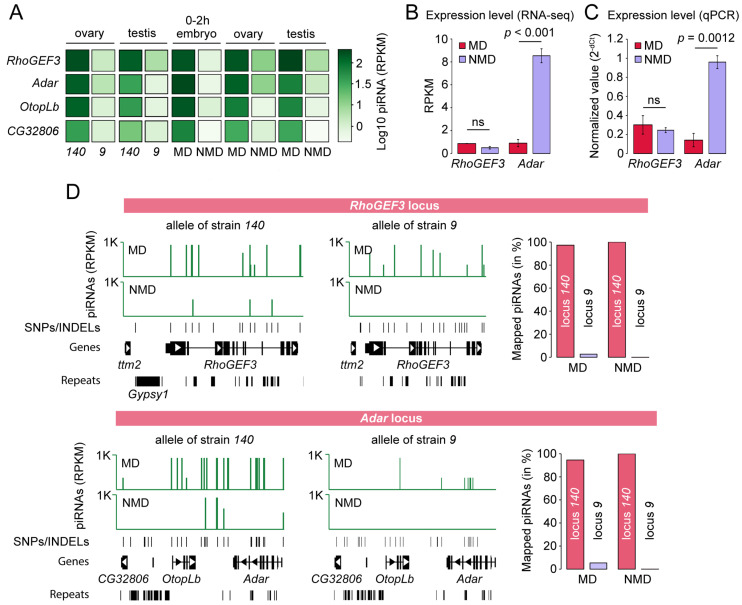
Maternal transmission of piRNAs drives their production in the progeny. (**A**) The effect of maternal transmission of piRNAs on piRNA processing. Expression of piRNAs in the gonads of strains *140* and *9*, as well as in MD (maternal deposition) progeny (females *140* crossed with males *9*) that inherited piRNAs derived from gene transcripts from their mothers and NMD (no maternal deposition) progeny (females *9* crossed with males *140*) that did not inherit these piRNAs. (**B**,**C**) Expression levels of *RhoGEF3* and *Adar* genes were determined by RNA-seq and qPCR, respectively. *p*-values for RNA-seq represent *p*-adjusted values and were calculated by differential expression analysis. Student’s *t*-test was used to compare the results of qPCR experiments. ns indicates not significant. (**D**) Allele-specific mapping of piRNAs in reciprocal hybrids. Only piRNA reads unambiguously mapped to SNPs and INDELs that are distinct between strains *140* and *9* are shown. Note that piRNAs are generated from the allele of strain *9* only in MD but not in NMD. The right panels show the number of piRNAs mapped to *14*0 and *9* alleles in MD and NMD progeny as a percentage of all mapped piRNAs to the coordinates of piRNA clusters in both strains. K indicates thousands.

The profiling of piRNAs originating from *RhoGEF3*, *Adar*, *OtopLb*, and *CG32806* gene transcripts showed higher piRNA expression in MD compared to NMD in both ovaries and testes (Figure 4A). Furthermore, an analysis of piRNAs was also conducted in 0–2 h old embryos laid by *140* and *9* mothers, constituting a sample of pure germline tissue. The analysis provided additional evidence that piRNAs originating from all the studied genes are present in germ cells and are transmitted maternally (Figure 4A).

To further evaluate the effect of piRNA production on gene expression, we performed poly(A) RNA-seq from the ovaries of MD and NMD progeny. We observed that *Adar* expression was significantly lower in MD compared to NMD progeny (*p*-adj < 0.001), indicating the correlation between the silencing of *Adar* expression and maternal transmission of *Adar*-derived piRNAs (Figure 4B). Just like in mothers, *RhoGEF3* expression in the offspring was not affected by piRNA production (Figure 2D and 4B). The difference in *Adar* expression and similarity in *RhoGEF3* levels in MD and NMD ovaries were further confirmed by qPCR (Figure 4C).

The identical genomes of the MD and NMD offspring contain two alleles each of the *RhoGEF3* and *Adar* loci, derived from parents *140* and *9*. Given that the genomes of both strains have been assembled, this provides an opportunity to study the impact of the maternally supplied piRNA on the expression of the individual alleles. To differentiate piRNAs mapped to *140* and *9* loci, we re-aligned piRNAs to diploid genome allowing only one location that piRNA read can align to with a perfect match (see Section 4 for details). This approach permits the unambiguous determination of the alleles of origin of the *RhoGEF3* and *Adar* loci and the estimation of the activity of piRNA clusters. We found that, apart from piRNA mapping to both *RhoGEF3* and *Adar* loci of strain *140* in MD progeny, piRNAs also aligned to alleles derived from the chromosomes of strain *9* (Figure 4D). Moreover, no piRNAs were aligned to analyzed alleles derived from the chromosomes of strain *9* in NMD progeny (Figure 4D). Aligned piRNAs to the *RhoGEF3* and *Adar* loci of chromosome of strain *9* constituted 2.7% and 5.4%, respectively, of the total pool of aligned piRNAs on both chromosomes in the MD progeny (Figure 4D). Note that this analysis was restricted to the region of uniquely mapped piRNAs to avoid the inaccurate mapping of reads.

Overall, in agreement with previous findings, we determined that the maternal transmission of piRNAs to the gene transcripts of *RhoGEF3*, *Adar*, *OtopLb*, and *CG32806* drives their production in the progeny in both *cis* and *trans* [27]. Moreover, the transgenerational inheritance of piRNAs stimulates piRNA generation from previously inactive homologous genomic loci. An analysis of the differential expression of genes targeted by piRNAs revealed that the expression of *Adar* is downregulated in the MD offspring. Given the identical genomes of reciprocal hybrids, this finding suggests that *Adar* expression is silenced by piRNAs inherited from *140* mother. In contrast, the expression of *RhoGEF3* remained unaffected by piRNAs in MD hybrids.

### 2.5. The piRNA Clusters Demonstrate Varying Capacity to Generate piRNAs in the Absence of the Original Inducer Alleles

The epigenetic conversion of a genomic locus due to paramutation in Drosophila includes the maternal transmission of piRNAs, which triggers piRNA production from the homologous locus in *trans* [28,29,30]. Previously, we showed that the *cdi* locus of *D. virilis* strain *160* exhibits paramutation-like behavior driven by the maternal deposition of homologous piRNAs [42]. Given that the maternal transmission of piRNAs originated from the *RhoGEF3* and *Adar* gene loci initiates piRNA production from naïve homologous alleles (Figure 4D), we decided to test these loci for their ability to be transcribed as piRNA clusters in subsequent generations in the absence of the original inducer alleles. Initially, to reduce the genetic polymorphism, isogenic strains from *140* and *9* stocks were developed by performing ten rounds of individual crosses. It was confirmed that the production of piRNAs from the *RhoGEF3* and *Adar* loci exhibited a consistent pattern across isogenic and stock fly strains (Supplementary Figure S5).

**Figure 5:**
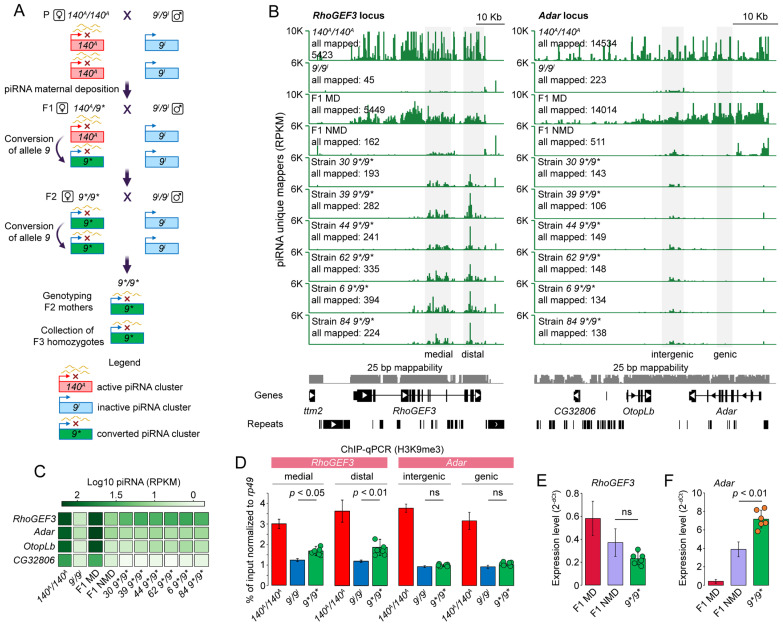
Testing for paramutagenic activity of piRNA clusters. (**A**) The system of piRNA-mediated silencing of *RhoGEF3* and *Adar* loci by conversion to a dual-strand piRNA clusters. The *RhoGEF3* and *Adar* loci in strain *140* produce piRNAs from both DNA strands. Results reported in this study indicate that maternally inherited piRNAs derived from both loci of strain *140* have the capacity to induce piRNA biogenesis from homologous alleles in strain *9* in *trans* (F1 in the figure). Paramutation describes an interaction between two alleles of a locus in which one allele (the paramutagenic allele) causes inherited epigenetic modifications in the other allele (the paramutable allele). The paramutation model in Drosophila is characterized by the maternal transmission of piRNAs, which enhance the production of piRNAs from homologous locus. Crucially, in subsequent generations, piRNA biogenesis can be maintained from epigenetically converted loci in the absence of the original triggering allele (9*/9* in the figure). (**B**) Coverage profile of uniquely mapped piRNAs to *RhoGEF3* and *Adar* loci (*D. virilis* genome r.1.06) in ovaries of strains *140* and *9*, F1 MD and NMD progeny as well as strains 9*/9* homozygous for *RhoGEF3* and *Adar* alleles of strain *9*. The number of all mapped shows all uniquely aligned and normalized piRNAs at a given locus. Gray areas demonstrate genomic regions that differentiate 9*/9* from strain *9* and NMD at the *RhoGEF3* locus, as well as genomic regions at the *Adar* locus used for ChIP-qPCR experiment. Unique mappability tracks for 25-bp-long reads are shown in grey. The repeat tracks depict the combined TEs and satellites data. K indicates thousands. (**C**) Expression of piRNAs derived from gene transcripts in parental strains, reciprocal hybrids, and progeny carrying 9*/9* alleles of *RhoGEF3* and *Adar*. (**D**) ChIP-qPCR analysis of H3K9me3 at the distinct regions of *RhoGEF3* and *Adar* loci in the ovaries of 9*/9* as well as parental strains *140* and *9*. The percentage of input was normalized to the actively transcribed *rp49* gene. The dots correspond to the values of each of the 9*/9* strains. Statistical significance was assessed using the Mann–Whitney U test. *RhoGEF3* medial amplicon corresponds to the genomic region scaffold_10322:438996-439095, *RhoGEF3* distal—scaffold_10322:446114-446273, *Adar* intergenic—scaffold_12942:65425-65551, *Adar* gene—scaffold_12942:76684-76817 in the genome of *D. virilis* r.1.06. (**E**,**F**) Expression levels of *RhoGEF3* and *Adar* genes, respectively, in ovaries of F1 offspring and progeny carrying 9*/9* alleles of *RhoGEF3* and *Adar*. Dots correspond to the values of each of the strains 9*/9*. Student’s *t*-test was used to compare the results of the qPCR experiment. ns indicates not significant.

The crossing scheme was designed as follows (Figure 5A): Strain *140* females (active piRNA clusters) were crossed with strain *9* males (inactive piRNA clusters). The resulting F1 hybrid females were then collected and backcrossed with strain *9* males (Figure 5A) (see Section 4 for details). The primary goal was to show that piRNA production can be maintained in backcross females (9*/9*) in the absence of the active piRNA cluster from the original allele of strain *140*. Once females backcrossed with strain *9* laid eggs, they were genotyped using an indel PCR assay to identify *RhoGEF3* and *Adar* alleles derived from strain *9* (Supplementary Figure S6, see also Section 4). Only the progeny from mothers homozygous for the *RhoGEF3* and *Adar* alleles of strain *9* were retained and used as strains 9*/9* for further analyses.

Upon completion of the experiment, we obtained six strains that met the specified genotype criteria (9*/9*, designated as strains *30*, *39*, *44*, *62*, *6*, and *84*). We first checked the piRNA profile at the *RhoGEF3* and *Adar* loci in the ovaries of the obtained strains 9*/9*. The results indicate that the aggregate number of uniquely mapped piRNAs to the *RhoGEF3* locus is significantly lower in 9*/9* strains in comparison to the F1 MD hybrids, where piRNAs were observed to be deposited from *140* mothers (Figure 5B). On the other hand, the piRNA profile of all examined 9*/9* strains demonstrated substantial changes compared to the F1 NMD hybrids, which lacked *RhoGEF3*-derived piRNA transmission from *140* mothers (Figure 5B). Specifically, we observed enhanced piRNA production from the medial and distal regions of the *RhoGEF3* gene relative to the distance to its transcription start site (Figure 5B). In contrast to the *RhoGEF3* locus, we failed to observe the increase in piRNA generation from the *Adar* locus of strains 9*/9* compared to F1 NMD (Figure 5B). In fact, the number of piRNAs originating from the *Adar* locus was even lower in strains 9*/9* than in the F1 NMD hybrids (Figure 5B).

The distal region of piRNA production in *RhoGEF3* spans the last few exons of the gene. To validate the increase in piRNA expression from *RhoGEF3* exons in strains 9*/9*, as demonstrated by piRNA genomic mapping, we further re-aligned piRNA reads to the *RhoGEF3* transcript. The analysis confirmed that the expression of piRNA to the *RhoGEF3* transcript increased approximately 6-fold in 9*/9* strains compared to F1 NMD progeny (Figure 5C). In contrast, no increase in piRNA expression targeting *Adar*, *OtopLb*, and *CG32806* mRNAs was observed (Figure 5C).

To verify that the increase in piRNA production is associated with a change in chromatin state at the *RhoGEF3* locus, we compared H3K9me3 mark levels using ChIP-qPCR performed on strains 9*/9* as well as on the parental strains *140* and *9*. For ChIP-qPCR, we designed primers to amplify parts of medial and distal regions of the *RhoGEF3* gene that show different piRNA generation capacities between strains 9*/9* and parental strain *9*. In agreement with the piRNA profile, the ChIP-qPCR analysis showed that both the medial and distal regions of the *RhoGEF3* gene were significantly enriched with the H3K9me3 mark in strains 9*/9* compared to parental strain *9* (*p* < 0.05 for medial, *p* < 0.01 for distal) (Figure 5D). To this end, the enrichment analysis of the H3K9me3 mark in two regions of the *Adar* locus was used as a control and demonstrated no difference between 9*/9* strains and strain *9* (Figure 5D).

By testing the effect of piRNA production on gene expression, we confirmed the previously established fact that piRNA targeting does not result in the silencing of *RhoGEF3* expression. The observed expression level of *RhoGEF3* showed no significant changes in 9*/9* strains compared to F1 NMD hybrids (Figure 5E). Surprisingly, we observed significantly elevated *Adar* expression levels in all 9*/9* strains compared to F1 NMD hybrids (Figure 5F). Probably, the silencing of the *Adar* allele derived from chromosome *140* occurs only *in cis* in F1 NMD. Thus, *Adar* is half expressed in F1 NMD and restored in 9*/9*, resulting in a ~2-fold increase in expression.

In summary, these data indicate that the *RhoGEF3* locus sustains piRNA production across subsequent generation (F3), even in the absence of the original inducer locus. However, the epigenetically converted *RhoGEF3* locus exhibits reduced piRNA expression in the absence of the original allele with an active piRNA cluster (Figure 5B). This can likely be attributed to the lack of *Gypsy1* insertion, which resulted in a greater number of piRNAs targeting this locus on the original allele. In contrast, piRNA expression from the *Adar* locus was not maintained in the offspring that lacked the original piRNA-producing allele.

## 3. Discussion

The piRNA pathway has evolved as a conserved, robust mechanism that regulates TE activity in the metazoan germline (reviewed in [51]). Although the majority of piRNAs are designed for TE silencing, numerous studies have demonstrated significant off-target effects of the co-transcriptional silencing of TEs that lead to gene repression [39,52,53,54].

In this paper, we extend the previous observation of the differential ability of genomic loci to generate piRNAs in *D. virilis* strains [13,40] and provide a detailed characterization of two genomic loci that produce genic piRNAs. Specifically, we demonstrated that four protein-coding genes, including *RhoGEF3*, *Adar*, *OtopLb*, and *CG32806*, operate as a source for piRNA production in the germline of strain *140* but not strain *9*. Following the identification of LTR retroelement insertion upstream of the *RhoGEF3* gene promoter, it is possible to reconstruct the events that likely led to the epigenetic conversion of this locus into a piRNA-producing site. The insertion of *Gypsy1* followed by the co-transcriptional silencing of the TE by piRNAs apparently initiated the deposition of the H3K9me3 mark, which spread downstream from the TE insertion to the neighboring *RhoGEF3* gene region. Eventually, the H3K9me3 mark at this locus was recognized by the chromodomain of the Rhino protein, a germline paralog of HP1, resulting in the formation of the dual-strand piRNA cluster.

Recent studies showed that complex satellite DNAs (>100 bp in length) in *D. melanogaster* are transcribed into long non-coding RNAs (lncRNAs) and processed into piRNAs [55,56]. The production of piRNA from satellite DNA depends on the Rhino–Deadlock–Cutoff complex and the transcription factor Moonshiner, a previously described non-canonical pathway that licenses the heterochromatin-dependent transcription of dual-strand piRNA clusters in the Drosophila germline [19,22,56]. These satellite DNA-derived piRNAs are detected in both ovaries and testes, suggesting a conserved regulatory mechanism across sexes [55,56]. Our analysis further demonstrated the proximity of the *RhoGEF3* and *Adar* gene regions to the *pvB370* (370 bp in length) satellite array towards telomeres. This satellite is specific to the *virilis* species group and shows a high degree of similarity to another 36 bp repeat of *D. virilis*, *pDv* [57,58]. Intriguingly, *pvB370* satellite is targeted by abundant sense and antisense piRNAs in both studied strains. In the Drosophila germline, the transcription of piRNA clusters is inextricably linked to the convergent transcription of genomic loci producing sense and antisense transcripts [59,60]. While we are yet to ascertain the origin of these piRNAs from this locus, it is nevertheless possible that these piRNAs are involved in the formation of the piRNA cluster in strain *140*. If this is indeed the case, it is conceivable that the transcription of *Gypsy1* on one side and the *pvB370* satellite on the other provided the necessary convergent transcription and led to the expansion of the piRNA cluster to the *RhoGEF3* gene in strain *140*. The present analysis did not reveal any significant structural differences that could underlie the formation of the piRNA cluster at the *Adar* locus. However, we cannot rule out that structural differences lie upstream of the *Adar* gene due to the incompleteness of the current genome assembly.

It is noteworthy that the reference genome of *D. virilis* sequenced during Drosophila 12 genomes program [46] and utilized for mapping of NGS data in this study also contains an LTR retrotransposon insertion at the *RhoGEF3* locus, albeit from a distinct family, *Tv1* (Figure 2A). One may suggest that the dual-strand piRNA cluster may also have been formed in this strain as well. The original fly stock used to create strain *15010-1051.87*, which was sequenced as part of this program, is believed to be strain *149*, which was developed at the Institute of Developmental Biology in Moscow (see Supplementary Material in [46]). Despite the absence of the opportunity to analyze this particular strain of *D. virilis* in the context of this study, the analysis of the piRNA profile for the derivative strain *160* was reported previously in a number of papers in the course of the investigation of hybrid dysgenesis syndrome [13,40,61]. Indeed, as indicated by the production of sense and antisense piRNAs from the *RhoGEF3* locus, the process of establishment of dual-strand piRNA clusters has been observed [13,27,40]. Unexpectedly, we failed to identify *Tv1* insertion along with any other TE insertions located in the region between *RhoGEF3* and *ttm2* genes in strain *160*. These data suggest that the *Tv1* insertion was probably lost during the breeding of this strain. This also suggests that the *RhoGEF3* locus may continue to function as a dual-strand piRNA cluster even in the absence of a causative mutation. As an alternative hypothesis, the TE insertion may not be the only factor underlying the epigenetic conversion of this genomic region.

In our investigation, we have witnessed both negative and positive correlations between the gene expression and corresponding antisense piRNA production in *D. virilis* genome. Thus, we determined that *Adar* expression was reduced in the ovaries of strain *140* compared to strain *9*, as well as in their hybrid, when piRNAs targeting *Adar* were maternally transmitted. It is imperative to acknowledge that the promoter region and the initial pair of exons of the *Adar* gene are absent from the genome assembly of strain *140* conducted in the present study. Therefore, the diminished expression of the *Adar* gene in strain *140* may be attributable not only to piRNA silencing but also to an unidentified mutation that has resulted in impaired transcription of the gene. On the other hand, the decreased expression of *Adar* in the F1 hybrid upon the inheritance of the maternal piRNA clearly indicates that the *Adar* gene is repressed as an outcome of piRNA silencing. In contrast, we failed to reveal a reduction in the expression of the *RhoGEF3* mRNA, even though there is a high level of antisense piRNA generation from the same locus. One may suggest that piRNA targeting the *RhoGEF3* gene could stimulate its mRNA expression in strain *140*, but the mechanism underlying this effect remains unclear.

Evidence for the role of small RNAs including piRNAs in transgenerational silencing was provided by studies in Drosophila. In this species, the female deposits the majority of the proteins and transcripts necessary for early embryonic development through the cytoplasm of the oocyte [62]. In contrast, the male contributes its genome, yet deposits only centrioles as cytoplasmic components [63,64]. The maternal cytoplasmic inheritance of piRNAs underlies the paramutation phenomenon in Drosophila [28,65].

Given the evidence that maternally transmitted piRNA to both *RhoGEF3* and *Adar* loci stimulate the epigenetic conversion of homologous alleles in the chromosomes of strain *9* in F1 progeny, we investigated the paramutagenic activity of these loci. The performed experiment provides two lines of evidence for the paramutation-like behavior of the *RhoGEF3* locus. First, the overall piRNA expression to *RhoGEF3* in the progeny devoid of the original paramutagenic allele is significantly higher compared to the paramutable allele of strain *9*. Second, we showed that increased piRNA expression correlates with H3K9me3 enrichment. Notably, the expression level of *RhoGEF3* mRNA is extremely low in the ovaries of *D. virilis* (<1 RPKM in terms of RNA-seq). Since co-transcriptional silencing by piRNAs requires active transcription, we suggest that the efficiency of the epigenetic conversion of the *RhoGEF3* locus in the chromosome of strain *9* is limited by the low expression of the *RhoGEF3* gene. Thus, piRNA production in this case is less efficient compared to the same locus in strain *140*, in which the TE insertion apparently stimulates piRNA biogenesis. Unfortunately, neither a significant increase in the expression of piRNAs originating from the *Adar* locus nor a decrease in *Adar* gene expression was observed in offspring devoid of the original paramutagenic allele from *140* mothers. Based on this observation, it can be concluded that the paramutagenic activity of the *Adar* locus is low and is not retained in the offspring if the original inducer allele is absent.

What drives the epigenetic conversion of the loci in the course of paramutation? Studies of paramutation in both plants and animals imply that small non-coding RNAs are the driver factor for the epigenetic conversion of the loci (reviewed in [29,30]). One may suggest that, in Drosophila, the paramutation would depend on the presence and stability of piRNA silencing as well as the activity of germline piRNA clusters. Indeed, on the one hand, the nature of germline piRNA clusters implies their paramutagenic behavior [66]. On the other hand, the variability in the capacity of piRNA clusters to manifest paramutagenic activity remains an area that has not yet been thoroughly investigated. The production of piRNAs in the Drosophila germline is orchestrated by Rhino and the histone methyltransferase Eggless/SETDB1, the enzyme responsible for the deposition of the H3K9me3 mark [20,23]. Deficiency in only one of these proteins results in the loss of piRNAs in germ cells. However, the mechanisms by which Rhino distinguishes piRNA clusters from the rest of the genome covered by H3K9me3 continues to be a major issue in the field. Intriguingly, a recent paper by Akkouche et al. revealed the role of dual histone modifications in determining Rhino-binding specificity [67]. Specifically, Rhino was demonstrated to bind to loci that were labelled not only with H3K9me3 but also with H3K27me3. In view of these points, it is only possible to make a tentative prediction of the paramutagenic activity of piRNA cluster until it is confirmed by experimental means.

Along with the previously described paramutation-like behavior of the *cdi* locus [42], the *RhoGEF3* locus represents another naturally occurring paramutagenic locus described in animals. Furthermore, akin to the *cdi* locus, the *RhoGEF3* and *Adar* loci are located in the subtelomeric region of chromosomes in *D. virilis*. Little is known about the telomere structure in *D. virilis*. Structural and functional analyses of *D. melanogaster* telomeres indicate the presence of three distinct regions of chromatin: a protective cap structure at the very end of the chromosome; a terminal array of retrotransposons of Jockey superfamily, including *HeT-A*, *TART*, and *TAHRE*, that alike other TEs are overseen by piRNAs; and telomere-associated sequences (TAS), which are composed of satellite-like repeats [68,69,70]. Telomeric and subtelomeric regions belong to a unique type of germline piRNA clusters. Transcripts originating from these piRNA clusters have been shown to function as both piRNA precursors and their sole targets [69]. Along these lines, in the *cdi* locus, the *TART* insertion is positioned in close proximity to the paramutagenic *cdi* allele, yet it is absent from the paramutable allele [42]. Furthermore, evidence was presented demonstrating that piRNAs targeting *TART* are more abundant in the strain that carries the paramutagenic *cdi* allele. It was hypothesized that this may contribute to the establishment of a *cdi* piRNA cluster in the inducer strain. In the *Adar* locus, another retroelement, *Uvir*, is located upstream of the *Adar* gene. This retroelement exhibits partial resemblance to *HeT-A* [71], which could provide a potential basis for generalizing the proximity of telomeric retroelements as a factor necessary to ensure the paramutagenic activity of the locus. However, *Uvir* was detected in strain *9*, but not in strain *140*, probably due to the incomplete current assembly of this strain. Moreover, the epigenetic conversion of the *Adar* locus was not preserved in the progeny in the absence of the original paramutagenic allele.

At the *RhoGEF3* locus, no proximity of the telomeric retroelement to the *RhoGEF3* gene was observed. Instead, *RhoGEF3* is located adjacent to a satellite array that is targeted by abundant piRNAs in both strains that have been studied. Moreover, the piRNA cluster at the *Adar* locus is flanked by satellite arrays, suggesting the involvement of satellite DNA in the establishment of the piRNA cluster. Studies on plants have demonstrated the importance of *cis*-regulatory sequences located upstream of the promoter region of paramutagenic and paramutable alleles for paramutagenic effect [72,73]. Which *cis*-regulatory regions operate paramutation in animals is unknown. It will be of interest to investigate the following hypotheses: first, whether the subtelomeric satellites of *D. virilis* are a source of piRNA production; second, if so, whether the combined effect of piRNA silencing of TE insertions and piRNA production from the satellite array affects the periodic appearance of dual-strand piRNA clusters spreading to neighboring gene regions, especially in the context of their potential paramutagenic activity.

Studies of the *cdi* and *RhoGEF3* loci have also demonstrated that telomeric repeat regions are shorter in strains carrying paramutagenic alleles. Consequently, the length and proximity of the capping proteins may be considered as contributing factors of paramutation.

## 4. Material and Methods

### 4.1. Fly Stocks and Husbandry

Two *D. virilis* strains were used: *9* (Batumi, Georgia) and the laboratory strain *140* (eb va). Fly stocks were obtained from the Stock Center of the Koltzov Institute of Developmental Biology RAS (Moscow, Russia). All flies were reared on a standard resin–sugar–yeast–agar medium at a constant temperature regime (25 °C).

### 4.2. Cross Scheme for Studying Paramutagenic Activity

To reduce genetic polymorphism, we developed isogenic strains from *140* and *9* stocks by performing ten rounds of individual crosses. From the resulting broods that produced viable and fertile offspring (eight for strain *9* and four for strain *140*), one brood for each strain with the highest fecundity was selected and retained as an isogenic strain for the experiment.

The general scheme for assessing paramutagenic activity was as follows: Females of isogenic strain *140* (carrying active piRNA clusters at *RhoGEF3* and *Adar* loci) were crossed with males of isogenic strain *9* (with inactive piRNA clusters at *RhoGEF3* and *Adar* loci). F1 hybrid females were collected and backcrossed with males of strain *9*. Subsequently, the female offspring were backcrossed again individually to males of strain *9*. Finally, we collected backcross progeny homozygous for the *RhoGEF3* and *Adar* alleles of strain *9* (strains 9*/9*).

Specifically, crosses between 50 virgin females of isogenic strain *140* and 25 males of isogenic strain *9* were conducted in vials containing 10 females and 5 males each. Among the F1 hybrids, 210 virgin females were collected and backcrossed with males of isogenic strain *9*. Subsequently, the female offspring (F2, 200 individuals) were subjected to an additional round of individual crosses with 2–3 males from isogenic strain *9*. Following a ten-day period, the parent males were removed from the vials, and the DNA was extracted from the F2 mothers for genotyping. Only offspring (F3) from mothers homozygous for the *RhoGEF3* and *Adar* alleles of strain *9* (genotype 9*/9*) were retained and used as strains for further analyses. We obtained six strains that satisfied the specified genotype criteria and analyzed the F3 generation in all subsequent experiments, including ChIP-qPCR, small RNA sequencing as well as RT-qPCR.

### 4.3. Genotyping Assay

A 69 bp deletion located between *Adar* and *OtopLb* genes in strain *9* was used to develop a genotyping assay for *Adar* loci of both strains. The distinct amplicon sizes between loci were easily distinguished from each other by agarose gel electrophoresis. The amplification of genomic region flanking the insertion of *Gypsy1* retroelement in strain *140* was theoretically the best option to develop genotyping assay for *RhoGEF3* alleles. However, despite the use of Taq and Encyclo DNA polymerases (Evrogen, Moscow, Russia), attempts to detect an amplicon longer than 5 kb in strain *140* were unsuccessful. Thus, we developed a genotyping assay for the *RhoGEF3* locus using two pairs of primers. The first primer pair allow to cover the region from the 3′ end of the *Gypsy1* insertion to the flanking downstream region, similar for both genomes. Utilizing this pair of primers, we observed a clear band for allele *140* and no signal for allele *9* after PCR amplification followed by gel electrophoresis. Using the second primer pair, we applied the developed strategy of utilizing genomic regions adjacent to the TE insertion. The application of these primers permitted the identification of a distinct band corresponding to allele *9* but not allele *140*. Sequences of primers used for the genotyping assay are given in Supplementary Table S2.

### 4.4. DNA Extraction, Library Preparation, ONT Sequencing, and Genome Assembly

An expansion of 7 bp satellite DNA in the pericentromeric and centromeric regions of all chromosomes resulted in the unusually large genome size of *D. virilis* in comparison to other Drosophila species (~390 Mb) [74]. In order to enrich the DNA library with euchromatic DNA sequences and thereby refine the quality of the genome assembly, we exploited the isolation of genomic DNA from ovarian tissue with under-replicated centromeric DNA, a consequence of polytenization [75].

Females of strain *140* collected for DNA extraction were aged for 10–15 days, starved for 6 h, then immediately subjected to genomic DNA isolation procedure. Genomic DNA was isolated from thirty pairs of ovaries using high-salt denaturing buffer containing 100 mM Tris-HCl (pH = 8), 10 mM EDTA (pH = 8), 350 mM NaCl, 2% SDS, and 7 M Urea following phenol-chloroform extraction. The size selection of high-molecular-weight DNA was performed using Short Read Eliminator Kit (Circulomics, Baltimore, MD, USA).

DNA libraries for sequencing were prepared using the 1D ligation SQK-LSK109 kit (Oxford Nanopore Technologies (ONT), Oxford, UK) following the recommended protocol from ONT using 1 µg of DNA. Sequencing was performed on a flow cell FLO-MIN-106D r9.4 (ONT, UK). Sequencing resulted in 10.5 billion bases with a median sequenced fragment length of 27.13 Kb.

The conversion of FAST5 to FASTQ format was performed with Guppy 6.4.6 using the super accurate flip-flop algorithm (dna_r9.4.1_450bps_sup.cfg). Quality filtering (Phred > 10) was completed by NanoFilt [76]. The adapter sequences were removed with Porechop [77]. Genome was assembled using Flye v2.9 (expected genome size was set to 200 Mb) [78]. Assembly quality was evaluated by the QUAST 5.1 [79] and the presence of universal single-copy orthologs by BUSCO software using BuscoDB version 4 with Diptera lineage [80]. For the reference-based QUAST assessment, we used the genome of D. virilis r.1.06 (fetched from https://flybase.org/ on 15 March 2020) assembled and annotated in the 12 Drosophila genomes project [46]. The genome annotation of *D. virilis* genome r.1.06 was transferred to other *D. virilis* genomes using Liftoff [81] with default parameters.

The genomic assembly of strain *140* is available at NCBI with the accession number PRJNA1234388. Raw ONT reads are available at NCBI SRA (SRX28458524). The genome of strain *9* was sequenced and assembled previously [48] and was obtained from NCBI (assembly GCA_016920725.1).

To pairwise-align genomic regions identified as piRNA clusters, we extended sequences 10 kb upstream and downstream from the ends of continuous piRNA mapping in assemblies of strains *140* and *9*. Alignment was performed using minimap2 [82] with preset option asm10. The resulting alignment file was visualized using dotPlotly (https://github.com/tpoorten/dotPlotly accessed on 27 April 2025)).

The mappability of reads with the genome sequence was assessed using the GenMap program [83]. The k-mer length was set to 25 for piRNA mapping and 50 for mRNA and ChIP sequencing.

### 4.5. RNA-Sequencing and Gene Expression Analysis

Total RNA for RNA-seq and small RNA-seq experiments (see below) was extracted from ovaries using Extract RNA reagent (Evrogen, Russia). The concentration of RNA was measured with a Qubit Fluorometer (Invitrogen, Carlsbad, CA, USA). The quality of RNA was determined with an Agilent BioAnalyzer 2100 using the RNA 6000 nano kit. The RNA integrity number (RIN) of all RNA samples taken for mRNA libraries preparation was not less than 8. Poly(A)-containing RNA was isolated using NEBNext Poly(A) mRNA Magnetic Isolation Module (New England Biolabs, Ipswich, MA, USA), and libraries for RNA-seq were prepared using the NEBNext Ultra II Directional RNA Library Prep Kit for Illumina (New England Biolabs, Ipswich, MA, USA) according to the manufacturer’s guidelines. Experiments were performed in two biological replicates for each *D. virilis* strain. Paired-end sequencing (50 + 50 nts) was conducted on an Illumina NextSeq 2000 platform (Illumina, Inc., San Diego, CA, USA).

The pre-processing of sequenced reads, including all NGS experiments, was performed using trim_galore (https://github.com/FelixKrueger/TrimGalore accessed on 27 April 2025), including adapter and quality (≥20 Phred) trimming. Trimmed RNA-seq reads were aligned to the *D. virilis* genome r.1.06 (https://flybase.org/) using STAR 2.7.1a [84]. Counts of RNA-seq reads mapped on gene transcripts were estimated using featureCounts [85] and normalized to transcript length as well as sequencing depth (RPKM, reads per kilobase per million mapped reads). Resulting values were considered as gene expression levels. Differential gene expression analysis was performed with DESeq2 [86].

The analysis of TE expression was conducted with TEtranscripts [87] using the TEs annotation file obtained with RepeatMasker [88] and converted to the gene transfer format (gtf). As a library of canonical TE sequences, we used the combined libraries from Repbase v27.01 [89] and the list of TEs collated in Erwin et al. [40]. Known satellite sequences including the *pvB370* [57], *pDv* [58], *154TR*, *172TR* [90], *225TR* [91] as well as abundant 7 bp [92] families present in the genome *D. virilis* were extracted from NCBI (GenBank ID for *pvB370*—Z29422.1, *pDv*—X03936.1) and the paper by Abdurashitov et al. [90]. The counts of RNA-seq reads mapped to the canonical TE sequences were normalized to TE length and sequencing depth (RPKM). The resulting values were considered as TE expression levels.

### 4.6. Quantitative PCR

Complementary DNA (cDNA) was prepared from 2 μg of RNA treated with Turbo-DNAase (Ambion, Austin, TX, USA) using oligo(dT) primer and MMLV revertase (Evrogen, Moscow, Russia). PCR analysis was performed on an ABI PRISM 7500 System instrument (Applied Biosystems, Waltham, MA, USA). Amplification products were detected using SYBR Green 1 with the addition of ROX dye (Evrogen, Moscow, Russia), according to the manufacturer’s protocol. Expression values were normalized to the *rp49* gene and calculated using the 2-dCt equation. The specificity of the amplified products was validated by Sanger sequencing and melt curve analysis. The resulting value of the expression level for each sample was determined based on three biological replicates. The applied primers for *RhoGEF3* and *Adar* genes were designed to span the exon–exon junction. The sequences of the primers used are shown in Supplementary Table S2.

### 4.7. Small RNA-Sequencing and piRNA Analysis

In the present study, we re-examined previously published small RNA-seq data. This includes small RNA-seq of ovaries, testes, and 0–2 h embryos of strains *140* and *9* and their reciprocal hybrids [13]. The data are available from the NCBI Gene Expression Omnibus with the accession number GSE22067.

For additional small RNA-seq, the ovarian small RNA fraction for the cloning of isogenic strains *140* and *9* and six strains 9*/9* was separated from total RNA (~15 μg) using 15% polyacrylamide gel electrophoresis containing 8M Urea. After incubation in an ethidium bromide solution (0.5 μg/mL), gel fragments corresponding to the small RNA fraction were excised, using chemically synthesized RNA corresponding to 20 and 29 nts as size markers. The cloning of small RNA libraries was performed using the Illumina TruSeq Small RNA prep kit (Illumina, Inc., San Diego, CA, USA), according to the manufacturer’s protocol.

Pre-processing included trimming for adapters, quality (≥20 Phred), and length (20–29 nt). Pre-processed reads were further subjected to subtraction of reads matching to all rRNA, tRNA, snRNA, and miRNA sequences of Drosophila genus. The selected reads were mapped to the reference genome (*D. virilis* r.1.06), assembled genome of *140* and *9*, as well as annotated gene transcripts of *D. virilis* by Bowtie [93], allowing up to 3 mismatches, retaining only uniquely mapped reads (tags: --all --tryhard -v 3 -m 1 --best --strata --quiet). We considered small RNA fractions of 23–29 nt in length as piRNAs. The analysis of piRNAs, including the counting of piRNAs, the sorting of sense and antisense piRNAs, and the calculation of ping-pong signatures was analyzed in accordance with the well-described technique [94] using custom scripts written in R and Python 3.6.12. Normalization to the number of reads per kilobase per million mapped reads (RPKM) was applied to obtain piRNA expression levels.

### 4.8. Allele-Specific Mapping of piRNA Reads

For allele-specific mapping, we created a diploid genome by merging genome assemblies of strains *140* and *9*. The mapping of piRNA reads was performed on a created bowtie index containing two versions of each chromosome (one for each strain) with the following parameters: --all --tryhard -v 0 -m 1 --best --strata --quiet, allowing only one location in the diploid genome that a read can perfectly map to. The counting of piRNA reads mapped to the defined piRNA clusters in both genomes were estimated using featureCounts [85] and normalized to the reads per kilobase per million mapped reads (RPKM).

Single nucleotide polymorphisms (SNPs) and insertions–deletions (indels) in the genomes of *140* and *9* within piRNA clusters were defined by pairwise alignment using Blastn algorithm [95].

### 4.9. ChIP-Seq

Chromatin from ovaries of strains *140* and *9* (~100 pairs for each IP experiment) was extracted and immunoprecipitated according to the published protocol [96]. ChIP experiments were carried out using commercially available antibodies anti-H3K9me3 (ab8898) (Abcam, Cambridge, UK). To bind antibodies, Pierce protein A/G agarose (Thermo Fisher Scientific, Waltham, MA, USA) was used. The construction of libraries for sequencing was performed using DNA Library Prep for Illumina (New England Biolabs, Ipswich, MA, USA). Experiments were performed in two biological replicates. Paired-end sequencing (50 + 50 nt) was conducted on an Illumina NextSeq 2000 platform. For the analysis of H3K9me3 enrichment in strain *9*, we used single-end sequencing data published previously [27] and deposited in NCBI GEO under the number GSE59965.

Trimmed sequenced reads were aligned to the reference genome (*D. virilis* r.1.06) using Bowtie2 [97]. Aligned reads were sorted by coordinates, filtered of most multi-mapped reads (samtools view -bh -q 10), and cleaned of duplicates (samtools rmdup) using SAMtools [98]. Filtered aligned reads were normalized using the RPGC method (number of reads per bin/scaling factor for 1× average coverage of the genome) implemented in deepTools2 package [99] with the command: bamCoverage --binSize 10 --normalizeUsing RPGC --effectiveGenomeSize (total length of the genome) --extendReads (for paired-end data). Next, the obtained values for input samples were subtracted from treat samples using the bigwigCompare script [99] resulting in coverage values for each sample. Aligned reads in bigwig format for ChIP-seq, RNA-seq, and small RNA-seq were visualized using the Integrative Genome Viewer (IGV) [100].

Peak calling was performed using MACS2 software [101] with the recommended parameters for narrow and broad peak calling as well as normalization on input chromatin controls.

### 4.10. ChIP-qPCR

Chromatin extraction and immunoprecipitation was performed as described above. The percentage of precipitated chromatin was calculated according to the input values followed by normalization to *rp49* gene. The resulting values represent the mean of two biological replicates for parental strains and the mean of all six strain 9*/9* obtained. Primer sequences are presented in Supplementary Table S2.

## 5. Conclusions

This study elucidates the paramutation-like behavior of genic piRNA-producing loci in *D. virilis*, highlighting the interplay between piRNAs, chromatin environment, and the structural features of subtelomeric regions. We demonstrated that maternally inherited piRNAs can epigenetically convert homologous loci into active piRNA clusters, with the *RhoGEF3* locus maintaining this activity across generations even in the absence of the original inducer allele, while the *Adar* locus fails to sustain piRNA production under the same conditions. These findings underscore the complexity of piRNA-mediated epigenetic regulation, which is influenced not only by small RNAs but also by the genomic and chromatin context. Our work provides new insights into the activity of subtelomeric piRNA clusters in Drosophila, contributing to a broader understanding of transgenerational epigenetic inheritance in metazoans.

## Data Availability

High-throughput sequencing data for ChIP-seq (strain *140*), RNA-seq, and small RNA-seq (isogenic strains *140* and *9* as well as six strains *9*/9**) experiments performed in this study are available through the NCBI GEO (accession number GSE292339). Raw ONT reads were deposited in the NCBI SRA (SRX28458524). Other reported small RNA-seq data (ovaries, testes and 0–2 h embryos of strains *140* and *9*, ovaries, and testes of reciprocal hybrids) were deposited in GEO under the number GSE22067. ChIP-seq for strain *9* can be obtained from GEO using accession number GSE59965.

## Supplementary Materials

The following supporting information can be downloaded at: https://www.mdpi.com/article/10.3390/ijms26094243/s1.
