# Supplementary material for: Paramutation-Like Behavior of Genic piRNA-Producing Loci in Drosophila virilis
Source: Int J Mol Sci. 2025 Apr 29;26(9):4243. doi: 10.3390/ijms26094243 (PMC12072073; doi:10.3390/ijms26094243)
Supplement: Supplementary file 1 [file ijms-26-04243-s001.zip › Supplementary_figures_revised2.docx]

**Supplementary figures to the article**

**“Paramutation-like behavior of genic piRNA-producing loci in *Drosophila virilis*” by Alina V. Bespalova et al.**


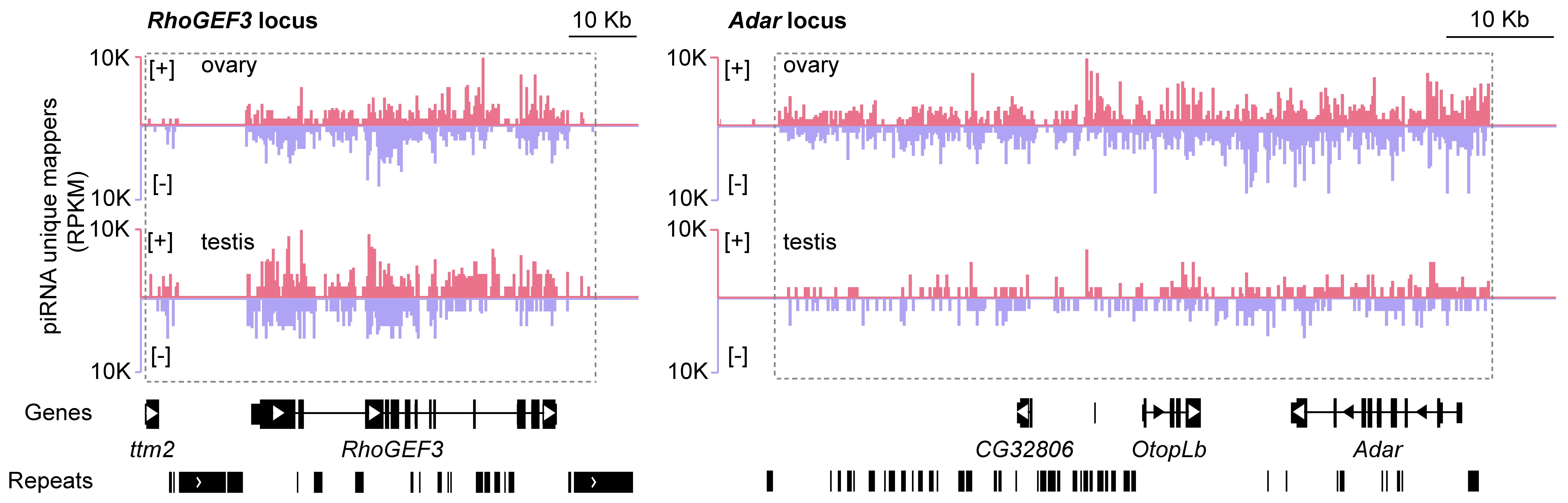


**Supplementary Figure S1.** piRNA mapping profiles of dual-strand piRNA clusters are similar in ovary and testis of strain *140*. Unique piRNA mappers are shown with respect to their mapping orientation to [+] and [-] DNA strand. Repeats track depicts the combined TEs and satellites data. K indicates thousands.


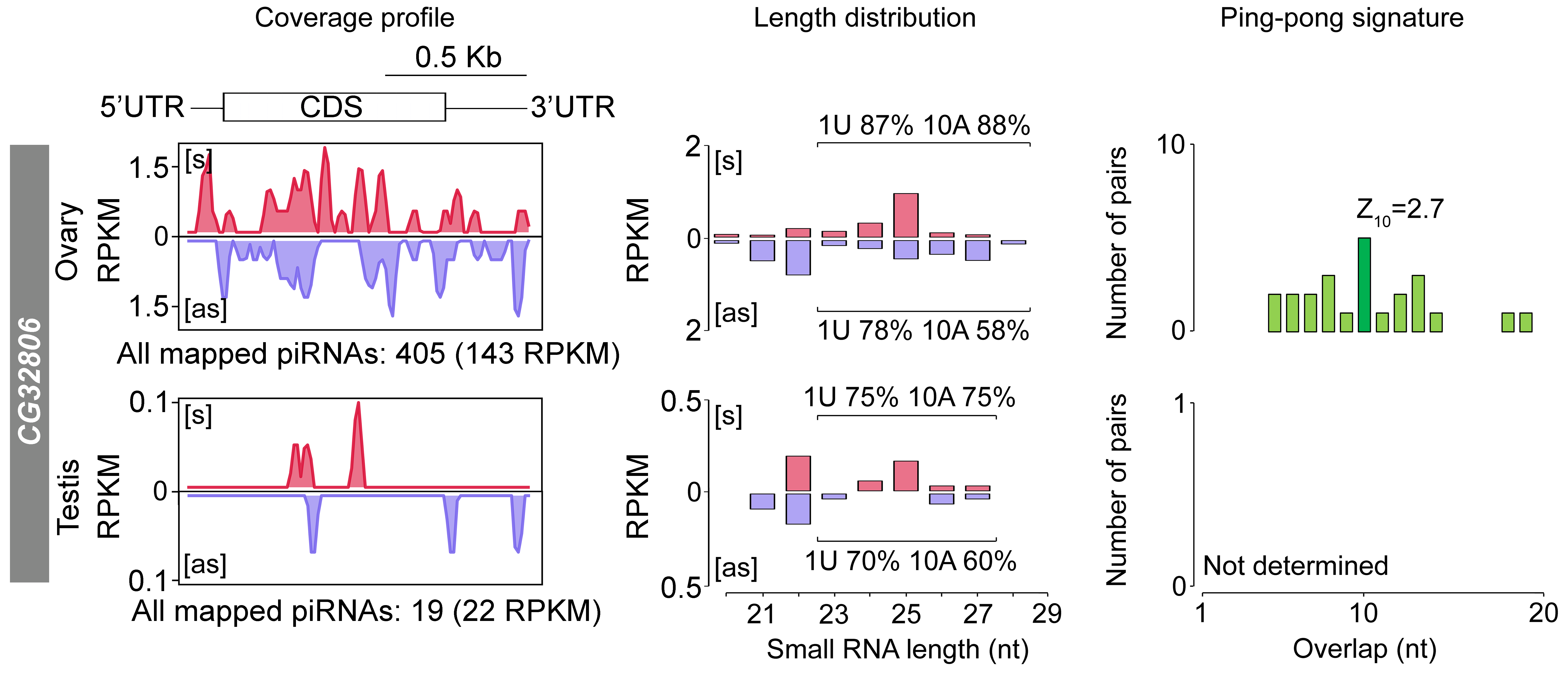


**Supplementary Figure S2.** Analysis of gene-derived piRNAs in ovaries and testes of *D. virilis* strain *140*. The left panels are profiles of mapped piRNAs (23-29 nts) for *CG32806* transcript in the two sexes. Only uniquely aligned piRNA reads with up to 3 mismatches are shown. Expression values were normalized to the number of reads per kilobase per million mapped reads (RPKM). Transcripts regions including open reding frame (ORF), 5’ and 3’ untranslated regions (5’UTR, 3’UTR) are shown above the plots. The number of all uniquely aligned piRNAs is shown below the plots. The middle panels - size distribution of uniquely mapped small RNAs 20-29 nt with up to 3 mismatches to each gene transcript. Nucleotide biases including 1U and 10A (percentage) are demonstrated for both sense and antisense piRNA mappers (23-29 nts). The right panels are the number of complementary piRNA pairs showing enrichment for 10 nt overlap between sense and antisense sRNAs (i.e. ping-pong signature). Ping-pong Z-scores are shown above the plots. S (red) and AS (blue) depict small RNAs mapped in sense and antisense orientation, respectively.


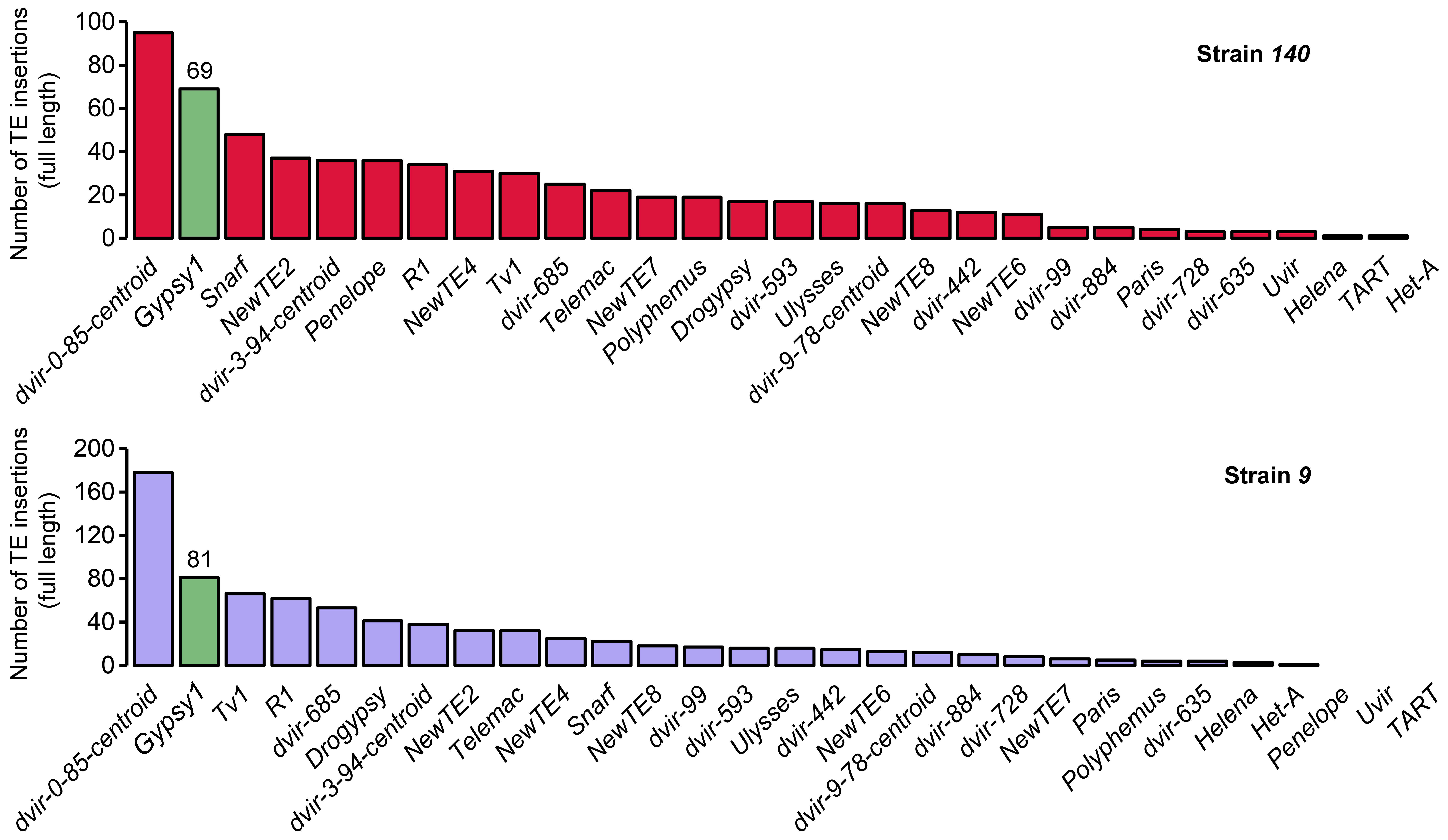


**Supplementary Figure S3.** The number of full-length TE insertions in genomes of *140* and *9*. TE insertions with coverage of at least 90% of the canonical TE length are considered full-length TE insertions.

**

**

**Supplementary Figure S4.** Comparative analysis of the *RhoGEF3* and *Adar* loci in the assembled genomes of strains *140* and *9*. (**A**) and (**B**) profiles of unique and all piRNA mappers for the *RhoGEF3* and *Adar* loci, respectively. TEs and satellites are shown with different tracks. Unique mappability tracks for 25-bp-long reads are shown in grey. The genomic regions of both strains are centred on the *RhoGEF3* gene locus and the *Adar* locus and are shown in grey. K indicates thousands.


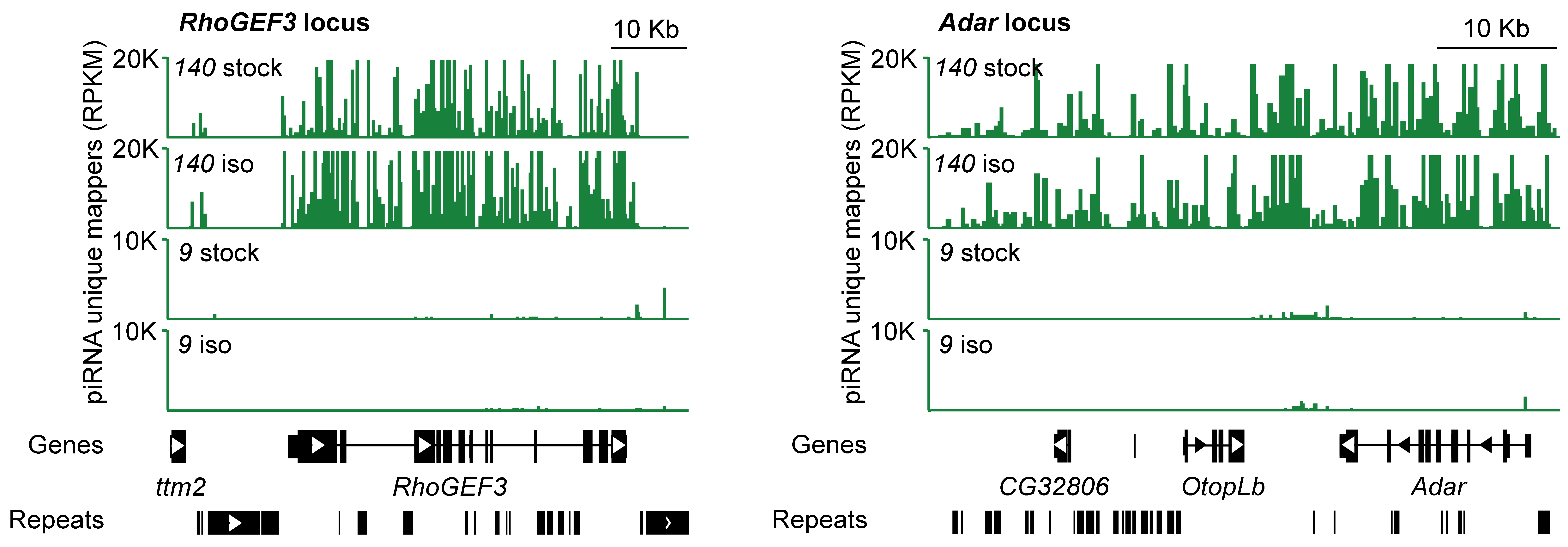


**Supplementary Figure S5.** Profiles of uniquely mapped piRNAs are similar between ovaries of the stock and isogenic strains *140* and *9*. Unique piRNA mappers are shown with respect to their mapping orientation to [+] and [-] DNA strand. Repeats track depicts the combined TEs and satellites data. K indicates thousands.


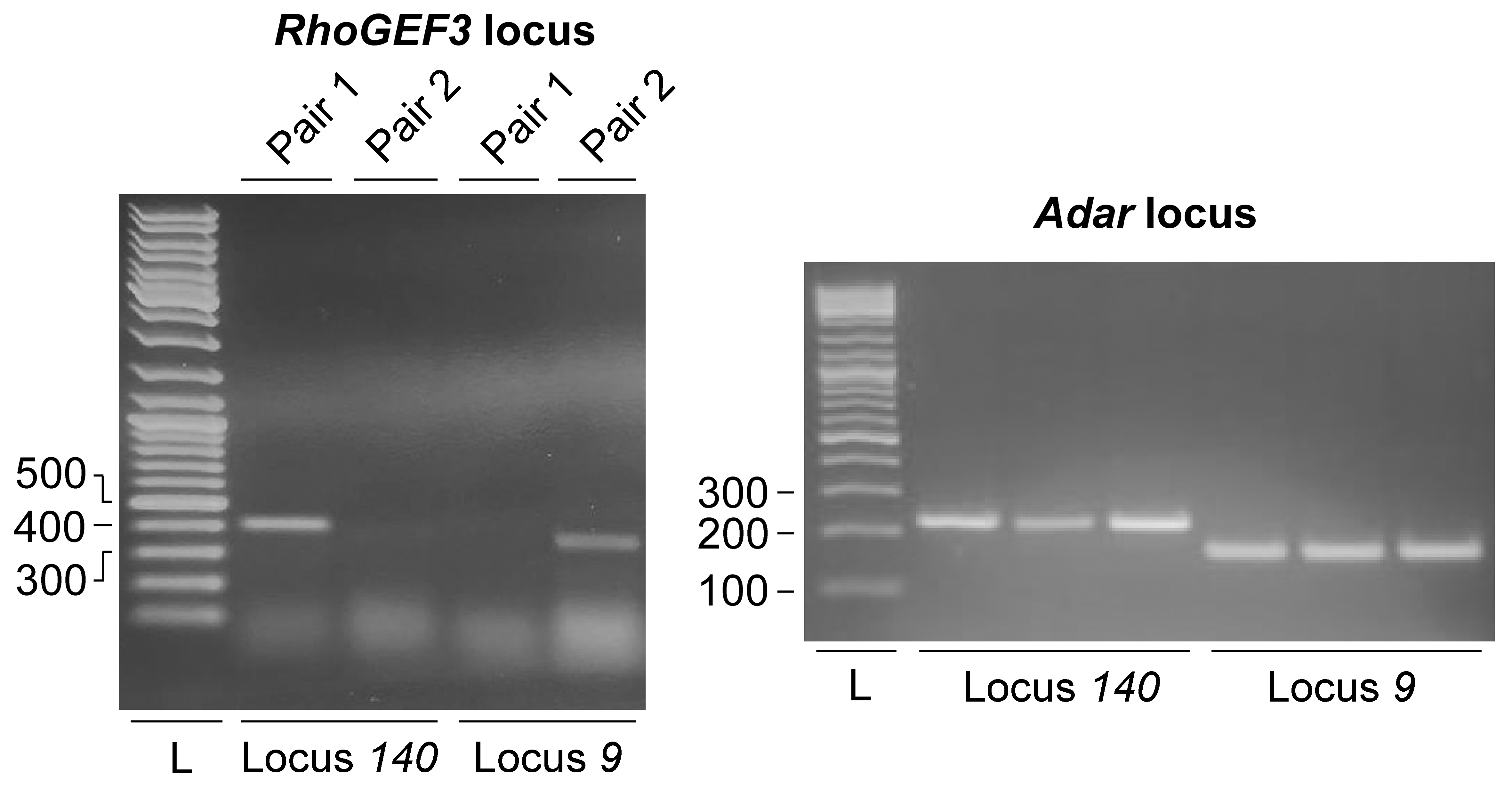


**Supplementary Figure S6.** Genotyping array. All the females that have produced offspring from backcrosses during the experiment for paramutation-like behavior were genotyped using an INDEL genotyping assay. Only offspring from mothers homozygous for the *RhoGEF3* and *Adar* loci of strain *9* were retained. For genotyping *RhoGEF3* locus we applied two pairs of PCR primers. The first pair of primers allows the identification of a distinct band corresponding to locus *140* but not locus *9*. The second pair of primers allows to observe a clear band for locus *9* but not locus *140*. A 69 bp deletion located between *Adar* and *OtopLb* genes in strain *9* was used to develop a genotyping assay for Adar loci of both strains. The analysis of strain *6* (genotype 9*/*9**) is presented as an example.
